# Interleukin-6 and indoleamine-2,3-dioxygenase as potential adjuvant targets for Papillomavirus-related tumors immunotherapy

**DOI:** 10.3389/fimmu.2022.1005937

**Published:** 2022-11-03

**Authors:** Roberta Liberato Pagni, Patrícia da Cruz Souza, Rafael Pegoraro, Bruna Felício Milazzotto Maldonado Porchia, Jamile Ramos da Silva, Luana Raposo de Melo Moraes Aps, Mariângela de Oliveira Silva, Karine Bitencourt Rodrigues, Natiely Silva Sales, Luís Carlos de Souza Ferreira, Ana Carolina Ramos Moreno

**Affiliations:** ^1^ Laboratório de Desenvolvimento de Vacinas, Instituto de Ciências Biomédicas, Departamento de Microbiologia, Universidade de São Paulo, São Paulo, Brazil; ^2^ ImunoTera Soluções Terapêuticas Ltda., São Paulo, Brazil; ^3^ Laboratório de Desenvolvimento de Vacinas, Instituto Butantan, São Paulo, Brazil

**Keywords:** HPV, immunotherapy, IDO, IL-6, vaccine, cancer

## Abstract

High-risk Human papillomavirus (HPV) infections represent an important public health issue. Nearly all cervical malignancies are associated with HPV, and a range of other female and male cancers, such as anogenital and oropharyngeal. Aiming to treat HPV-related tumors, our group developed vaccines based on the genetic fusion of the HSV-1 glycoprotein D (gD) with the HPV-16 E7 oncoprotein (gDE7 vaccines). Despite the promising antitumor results reached by gDE7 vaccines in mice, combined therapies may increase the therapeutic effects by improving antitumor responses and halting immune suppressive mechanisms elicited by tumor cells. Considering cancer immunosuppressive mechanisms, indoleamine-2,3-dioxygenase (IDO) enzyme and interleukin-6 (IL-6) stand out in HPV-related tumors. Since IL-6 sustained the constitutive IDO expression, here we evaluated the therapeutic outcomes achieved by the combination of active immunotherapy based on a gDE7 protein-based vaccine with adjuvant treatments involving blocking IDO, either by use of IDO inhibitors or IL-6 knockout mice. C57BL/6 wild-type (WT) and transgenic IL-6^-/-^ mice were engrafted with HPV16-E6/E7-expressing TC-1 cells and treated with 1-methyl-tryptophan isoforms (D-1MT and DL-1MT), capable to inhibit IDO. *In vitro*, the 1MT isoforms reduced IL-6 gene expression and IL-6 secretion in TC-1 cells. *In vivo*, the multi-targeted treatment improved the antitumor efficacy of the gDE7-based protein vaccine. Although the gDE7 immunization achieves partial tumor mass control in combination with D-1MT or DL-1MT in WT mice or when administered in IL-6^-/-^ mice, the combination of gDE7 and 1MT in IL-6^-/-^ mice further enhanced the antitumor effects, reaching total tumor rejection. The outcome of the combined therapy was associated with an increased frequency of activated dendritic cells and decreased frequencies of intratumoral polymorphonuclear myeloid-derived suppressor cells and T regulatory cells. In conclusion, the present study demonstrated that IL-6 and IDO negatively contribute to the activation of immune cells, particularly dendritic cells, reducing gDE7 vaccine-induced protective immune responses and, therefore, opening perspectives for the use of combined strategies based on inhibition of IL-6 and IDO as immunometabolic adjuvants for immunotherapies against HPV-related tumors.

## Introduction

Human papillomavirus (HPV) is the most common cause of sexually transmitted illnesses worldwide (1). Nearly all cervical malignancies and a range of other female and male cancers, such as anogenital and oropharyngeal, are associated with high-risk HPV, especially HPV-16 and HPV-18. Despite the preventability of HPV-related malignancies by prophylactic vaccines, there is still a high global incidence, particularly in low- and lower-middle-income countries. In this scenario, cervical cancer is the ninth most prevalent cancer worldwide and the fourth in terms of incidence and mortality in women (2). The conventional treatment of cervical cancer depends on the extent of the disease and fertility-sparing, which may include surgery, radiotherapy, and/or chemotherapy (3–5). However, even following usual treatments, recurrence of cervical cancer is still prevalent (3, 6), emphasizing the need for novel curative antitumor approaches.

Therapeutic failure is mainly attributed to the systemic and local immunosuppression induced by the oncological disease, which depends on tumor and host factors and involves different inflammatory molecules (7). Interleukin-6 (IL-6) is one such inflammatory molecule produced by many cell types, including tumor cells. IL-6 plays a crucial role in the proliferation and differentiation of malignant cells and it is known to be implicated in the pathogenesis of HPV^+^ cervical cancer (8). Compared to the normal cervix and cervical intraepithelial neoplasia (CIN), the expression of IL-6 in cervical cancer was considerably higher (9). Furthermore, circulating IL-6 was found to be a risk indicator since elevated serum IL-6 levels correlate with advanced stages of cervical cancer (10, 11). Regarding immunomodulation, while IL-6 promotes the recruitment of myeloid-derived suppressor cells (MDSC) into the tumor microenvironment, it hampers Th1 lymphocytes infiltration (12, 13), and dendritic cells activation (14).

The autocrine activation of IL-6 is responsible for STAT3 phosphorylation in HPV-related malignancies, particularly in cervical cancer (15). Interestingly, the IL-6 signal loop on a self-sustaining IL-6/STAT3/AHR axis is one of the mechanisms that maintain the constitutive expression of the indoleamine 2,3-dioxygenase (IDO) enzyme in tumor cells (16). IDO has gotten attention as one of the many mediators of tumor immune escape (17), since it degrades the essential amino acid tryptophan, creating a tryptophan-deficient microenvironment with critical immunological outcomes. IDO-expressing dendritic cells mediate T-cell suppression and/or tolerance, while low tryptophan concentration reduces T-cell-mediated responses by inhibiting T-cell proliferation and activation (18, 19). Importantly, cervical cancer expresses one of the highest amounts of IDO (20–22), and both IL-6 and IDO are negative prognostic markers in patients diagnosed with this neoplasm (8, 23), highlighting the IDO/IL6 axis as an important self-immunoregulatory network in cervical cancer. Consequently, blocking or inhibiting IL-6 signaling pathways may provide an interestingly therapeutic target to re-sensitize cancer cells to immunotherapies.

Regarding biotechnology breakthroughs, immunotherapy either based on passive administration of monoclonal antibodies or active immunization with vaccines has become a powerful ally to fight cancer. Immuno-oncological treatments aim to boost the immune system to recognize and attack cancer cells, as well as target immunosuppressive checkpoints to restore an immunological effector milieu (24). Over the last years, our group developed vaccines based on genetic fusions of HSV-1 glycoprotein D (gD) and HPV-16 oncoproteins aiming to treat HPV-related tumors and demonstrated the promising antitumor effects associated with combined adjuvant therapies in tumor-bearing mice (25–27). Our present study reports the testing of therapeutic adjuvant strategies focusing on IL-6 and IDO in combination with a protein-based (gDE7) antitumor vaccine.

## Material and methods

### Tumor cell line and culture conditions

The TC-1 cell line (28) was kindly provided by Dr. T.C. Wu from John Hopkins University in Baltimore, MD, USA. The TC-1 cells were cultured as previously described (27) and harvested at 90% confluency for subculture procedures, *in vitro* experiments, and *in vivo* assays. As a quality control, the expression of the oncoprotein E7 was confirmed by RT-PCR (data not shown), and cells were frequently tested for the absence of *Mycoplasma* spp.

### IL-6 secretion and gene expression assays

The TC-1 cells were seeded in 6-well cell dishes (1,5x10^5^/well) and cultured in RPMI 1640 medium supplemented with 10% of fetal bovine serum (FBS) (R10) until reaching 50-60% confluency. Next, cells were treated with a fresh R10 medium containing 1mM of 1-methyl-D-tryptophan (D-1MT), 1-methyl-L-tryptophan (L-1MT), or 1-methyl-DL-tryptophan (DL-1MT) and incubated for 24 hours at 37°C and 5% CO_2_. In the control group, only R10 medium was added. Cell culture supernatant was collected for cytokine measurement by BD™ Cytometric Bead Array (CBA) kit (#560485, BD Biosciences). Stained samples were acquired by LSR Fortessa™ (BD Biosciences) flow cytometer and data were analyzed using FlowJo software (TreeStar).

### Mice strains and tumor cell line implantation

Female wild-type (WT) C57BL/6 mice (6-8 weeks old) were purchased from the Faculty of Veterinary Medicine and Zootechnics of the University of São Paulo (USP). Male or female (on demand) IL-6 gene knocked mice (IL-6^-/-^) were supplied by the animal facility unit of the Department of Immunology of the University of São Paulo. Animals were allowed free access to water and food and provided with a 12h light/dark cycle, at 20-26°C temperature. Mice experiments were performed under approved protocols by the ethics committee for animal experimentation (protocol number CEUA 8572030918) and followed the standard rules approved by the National Council for Control of Animal Experimentation (CONCEA). The TC-1 cells were harvested at 90% confluency and transplanted into mice as previously described (27), at a concentration of 1x10^5^ cells/100µL/animal on day 0 (D0). Mice were considered tumor-bearing when tumors became palpable (7-10 days) and were euthanized when tumors reached 15mm in diameter or if they showed signs of distress (grimace scales).

### Mice gDE7-based immunotherapy and IDO inhibitors (1MT) treatment

**Figure 1:**
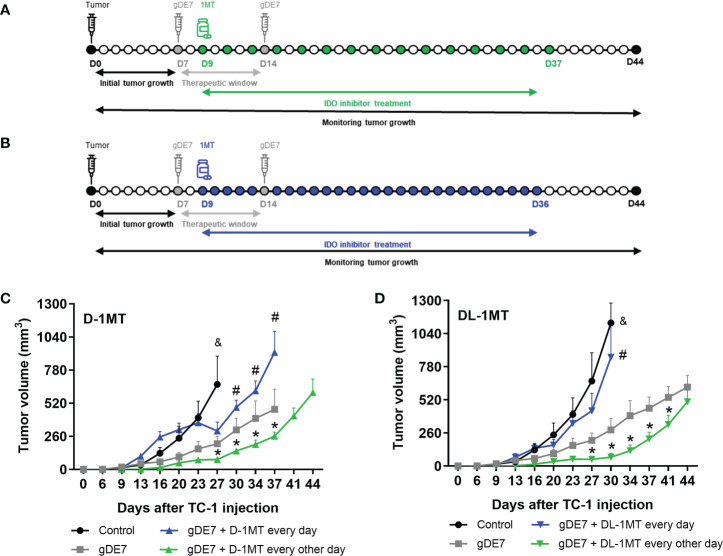
The administration regimen of 1MT isoforms affects the therapeutic antitumor effects of gDE7. Female WT mice were inoculated with TC-1 (D0) cells. Seven (D7) and fourteen (D14) days after the tumor engraftment, the animals were immunized with gDE7 (30μg, subcutaneously). Two days after the first dose (D9), mice were treated with 1MT isoforms **(A, B)**, either daily with 8mg/animal **(A)** (n = 10) or 10 mg/animal every other day **(B)** (n = 10) for four weeks, until day 36 (D36) or day 37 (D37), respectively. The experimental groups were followed for 60 days. Tumor volumes were followed up to 44 days in mice treated with the different treatments using **(C)** D-1MT or **(D)** DL-1MT. The tumor growth “endpoint data” for each group was plotted up to the date when at least 80% of the mice were alive. The data represent the average of two independent experiments and were analyzed by ANOVA. (&) p <0.05, statistical significance of control group concerning all the others; (#) p <0.05, statistical significance of gDE7 + 1MT every day group concerning all the others; (*) p <0.05, statistical significance of gDE7 + 1MT every other day group concerning all the others. The results were confirmed through multiple comparisons by Turkey’s test (three or four groups) or Sidak’s test (two groups), comparing each group mean with the other group mean at the same time point. D0 - day zero; D7 - day 7; D9 - day 9; D14 - day 14; D36 - day 36; D37 - day 37; D44 - day 44.

The therapeutic gDE7-based vaccine was administered following a regimen of two subcutaneous immunizations at a week interval, as previously described (27). Each dose contained 30 µg of the gDE7 protein, diluted in PBS, in a total volume of 100 µL, and inoculated at the right rear flank region of mice. Animals were immunized with gDE7 seven (D7) and fourteen (D14) days after TC-1 cell engraftment (D0). Treatment with oral administered D-1MT or DL-1MT began two days (day 9 - D9) after the first gDE7 immunization and lasted four weeks until day 36 (D36) for mice treated every day with 1MT (**Figure 1A**) or until day 37 (D37) for mice treated every other day with 1MT (**Figure 1B**). The D-1MT and the DL-1MT were administered to the animals at 8mg animal^-1^ every day or 10 mg animal^-1^ every other day, dissolved in a mixture of 0.5% tween-80, 0.5% methylcellulose in sterile Milli-Q water, being administered 100µL/animal per gavage.

### The antitumor effect assessment

The single or combined treatment outcome was evaluated by tumor mass volume, mice survival, and tumor-free mice. Tumor volume was plotted up to day 44 (D44). The “monitoring endpoint” of each group could be different since we chose as the “endpoint data” a survival rate of at least 80% of each mice group. Both survival and tumor-free mice were assessed up to day 60 (D60). The formula 1/2 [(length)^2 × width] was used to determine tumor volume.

### Intracellular cytokine staining

Intracellular IFN-γ staining was performed in peripheral blood samples collected in heparin-containing vials 7 days (day 21 – D21) or 14 days (day 28 – D28) after the last gDE7 immunization (D14). Cells were treated with ACK lysing buffer to remove red blood cells. The lysis was quenched by adding an R10 medium to the samples. Immunological cells were incubated overnight at 37°C and 5% CO_2_ in 96-well U-bottom plates (Corning) in RPMI 1640 medium supplemented with 10% FBS, 2 mM L-glutamine, 1 mM sodium pyruvate (Gibco), 1% non-essential and essential amino acid solution (v/v) (Gibco), 1% vitamin solution (v/v), in the presence of brefeldin A 10 μg mL^-1^ (GolgiPlug BD Biosciences), IL-2 (5 ng mL^-1^; Sigma), with or without the stimulation of the CD8-specific E7 peptide (^49^RAHYNIVTF^57^; GeneScript; 1,5μg/mL (29). After the incubation period (10 to 12 hours), cells were stained with anti-CD8-APC mAb (#553035, Biolegend), fixed, and permeabilized using Cytofix/Cytoperm kit (#555028, BD Biosciences), and stained with anti-IFN-γ-PE mAb (#5058808, Biolegend). Finally, samples were acquired on an LSR Fortessa flow cytometer (BD Biosciences) and analyzed using the forward scatter (FSC)/side scatter (SSC) parameters for the doublet exclusion gate, following the percentage of CD8^+^ IFN-γ^+^ T lymphocytes. The data were analyzed by FlowJo software (Tree Star).

### Tumor microenvironment immune cells analyses

For WT and IL-6^-/-^ tumor-bearing mice, tumors were collected 21 post tumor cell transplantation (D21), and cells were recovered by digesting the tumor mass with 22 U mL^-1^ of collagenase D (#11088866001, Roche Diagnostics) for 1h at 37°C, gently stirring every 10 min. After the incubation period, the collagenase was inactivated with 5 mM EDTA at room temperature for 5 min. The samples were gently resuspended in R10 and filtered through 70 μm cell strainers (Easy Strainer Greiner Bio-One). After centrifugation (300 g for 10 min), the pellet was resuspended in R10, filtered through 40 μm cell strainers, pelleted once again, resuspended in PBS containing 2% FBS, and distributed in 96 well U-bottom plates for further staining. The following mAbs were used to discriminate different subtypes of immune cells: anti-CD45-PerCP-Cyanine 5.5 (#103131, BioLegend), anti-CD4-FITC (#130308, BioLegend), anti-CD4-BV605 (#100451, BioLegend), anti-CD8-APC (#100712, BioLegend), anti-CD11b-Alexa Fluor 700 (#101222, BioLegend), anti-Ly6C-FITC (#128006, BioLegend), anti-Ly6G-PE (#127608, BioLegend), anti-Gr1-PE (# 553128, BD Pharmingen), anti-CD11c-PE (#553802, BD Pharmingen), anti-CD25 (#12-0251-82, eBioscience), anti-FoxP3-PE (#560414, BD Pharmingen), and anti-MHC-II-FITC (#107606, BioLegend). For the analysis of immune cell activation, anti-CD86-BV605 (#105037, BioLegend) was used. Cells were characterized according to the following parameters: T cells (CD45^+^, CD4^+^ or CD8^+^), dendritic cells (CD45^+^, CD11c^high^, MHC-II^high^), inflammatory monocytes (CD45^+^, CD11b^int^, Ly6C^high^, Ly6G^-^), resident monocytes (CD45^+^, CD11b^int^, Ly6C^int^, Ly6G^-^), polymorphonuclear myeloid-derived suppressor cells (PMN-MDSC) (CD45^+^, CD11b^high^, Ly6C^int^, Ly6G^+^ or Gr1^high^ CD11b^+^), T regulatory cells (Treg) (CD4^+^, CD25^+^, FoxP3^+^), and E7-specific CD8^+^ IFN-^+^ T cells (CD8^+^, IFN-γ^+^). Cells were acquired by LSR Fortessa™ (BD Biosciences) flow cytometer and data were analyzed using FlowJo software (TreeStar).

### Statistical analysis

Statistical analyses were performed using GraphPad-Prism software. The analysis was performed using the unpaired T-test, One-Way ANOVA, or Two-Way ANOVA and the results were confirmed through multiple comparisons by Turkey’s test or Sidak’s test, according to the GraphPad-Prism software recommendation. Survival curves were compared using the log-rank (Mantel-Cox) test. Appropriate methods were indicated in the legends. Values of p < 0.05 were considered significant.

## Results

### Mice treated with gDE7 and 1MT isoforms promote partial tumor mass control according to the administration regimen

**Figure 2:**
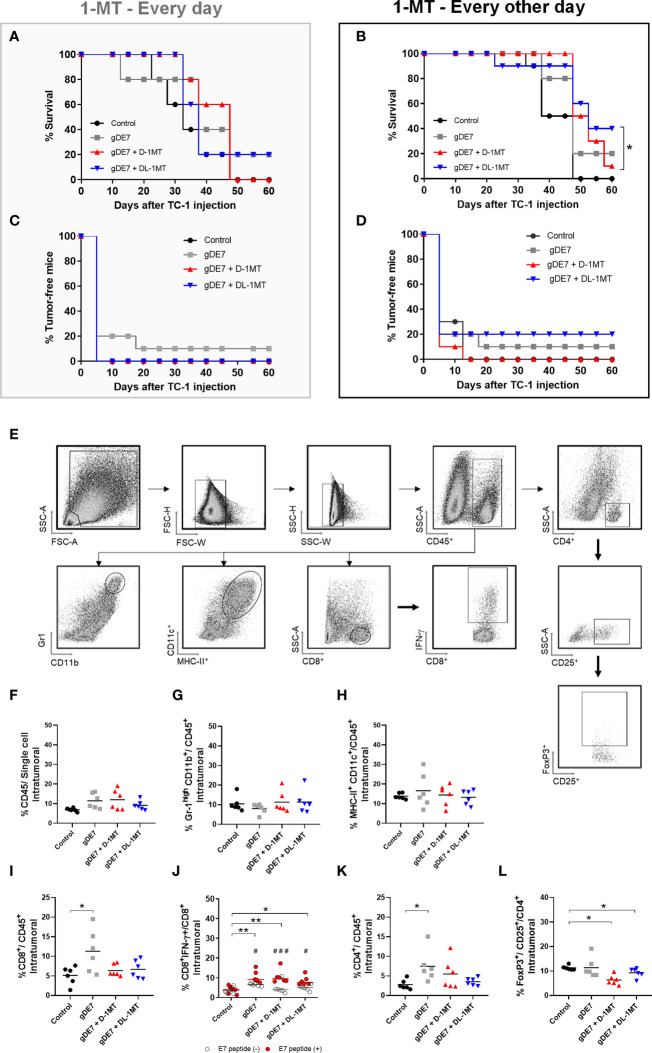
The combination of gDE7 and 1MT improves mice survival depending on their administration regimen. **(A–D)** The data represent the average of two independent experiments (n=5, total n=10). **(A, B)** Survival curves. Data were analyzed by Kaplan-Meyer test. **(A)** Two mice from gDE7 group and from gDE7 + DL-1MT survived until day 60. **(B)** Two, one, and four mice from gDE7, gDE7 + D-1MT, and gDE7 + DL-1MT groups survived until day 60, respectively. **(C, D)** Tumor-free mouse curves. Data were analyzed by ANOVA test* p <0.05. **(E)** Gating strategy for immune cell analyses in the tumor microenvironment, evaluated at day 21 after the tumor engraftment. Doublets were initially excluded by FSC-H versus FSC-W parameters, followed by SSC-H versus SSC-W parameters. Cells were gated by the expression of CD45^+^ and successively analyzed for: CD8^+^, followed by CD8^+^ IFN-g^+^; CD4^+^, followed by CD25^+^ and FoxP3^+^ CD25^+^ (T regulatory cells); CD11c^high^ MCH-II^high^ (dendritic cells); and Gr1^high^ CD11b^+^. **(F)** Frequency of CD45^+^ cells. **(G)** Frequency of Gr1^high^ CD11b^+^ cells. **(H)** Frequency of CD11c^high^ MCH-II^high^ cells. **(I)** Frequency of CD8^+^ T cells. **(J)** Frequency of E7-specific CD8^+^ IFN-g^+^/CD8^+^ cells. (#) p<0.05 represent the statistical significance of stimulated (red dots) versus non-stimulated (white dots) cells inside each experimental group. **(K)** Frequency of CD4^+^ T cells. **(L)** Frequency of FoxP3^+^ CD25^+^ CD4^+^ cells. Data representative of two independently performed experiments (n=6). Statistical significance: (*) p<0.05, (**) p<0.01 by ANOVA. (#) p<0.05 (###) p<0.001 represents the ANOVA statistical significance of stimulated (red dots) versus non-stimulated (white dots) cells inside each experimental group.

In previous work, we showed that the combination of gDE7 with 1MT partially controls the growth of TC-1 cells in mice (27). Since gDE7 conferred complete antitumor protection in IDO^-/-^ knocked mice (27), here we compare two therapeutic regimens in wild-type mice with administration of 1MT isoforms every day (**Figure 1A**), and every other day (**Figure 1B**). Notably, administration of 1MT isoforms every day did not improve treatment outcomes (**Figures 1C, D**). On the other hand, administration of D-1MT or DL-1MT every other day improved the therapeutic antitumor effects of the gDE7-based immunotherapy (**Figures 1C, D**). Regarding mice survival (**Figures 2A, B**) and tumor-free outcome (**Figures 2C, D**), mice submitted to vaccination and treated with 1MT every other day (**Figures 2B, D**) outperformed the group that was treated every day (**Figures 2A, C**). Mice treated with DL-1MT showed higher survival rates (**Figure 2B**) and tumor-free conditions (**Figure 2D**). Importantly, when 1MT therapy (D-1MT or DL-1MT) is interrupted, there was a decline in tumor growth control (**Figures 1C, D**), as also indicated by the mice survival rate after day 40 (**Figures 2A, B**). Given that every other day treatment with IDO inhibitors had a better outcome, we proceeded the experiments using solely this treatment strategy. Therefore, we next investigate the tumor-infiltrating immune cells population (time point analyzed - D21) to evaluate the immunological mechanism triggered by the chosen therapeutic approach (**Figure 2E**). Mice immunized with gDE7 and those also treated with IDO inhibitors (D-1MT or DL-1MT) showed similar frequencies of CD45^+^ cells, PMN-MDSC and dendritic cells (**Figures 2F-H**). Regarding T cell population, although only gDE7 immunization induced higher rates of intratumoral CD8^+^ T cells (**Figure 2I**), immunization with gDE7 with or without IDO inhibitors leads to increased frequency of E7-specific CD8^+^ IFN-^+^ T cells (**Figure 2J**). Interestingly, higher rates of intratumoral CD4^+^ T cells were found in mice immunized with gDE7 (**Figure 2K**), but only the combined therapy was able to decrease Treg population (**Figure 2L**). These findings suggest that the antitumor effects of IDO inhibitors, when employed as immunometabolic adjuvants, depend on the administration regimen. Moreover, when combined with immunotherapy, IDO inhibitors have a beneficial effect on Treg tumor-infiltration.

### IL-6 expression promotes tumor growth and negatively impacts gDE7 vaccine efficacy

**Figure 3:**
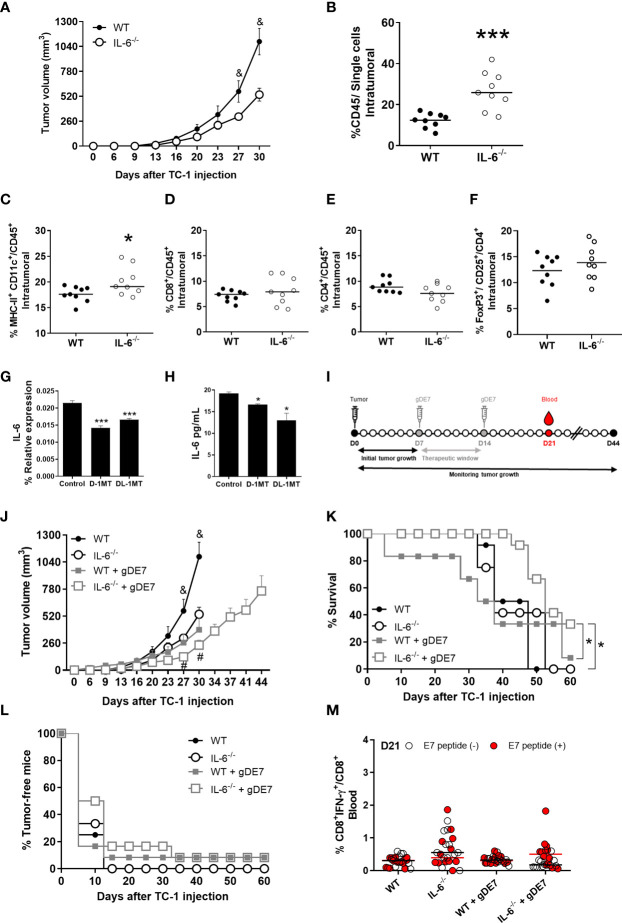
Expression of IL-6 affects immune cells and negatively impacts gDE7 antitumor effects. **(A)** Wild-type (WT) and IL-6^-/-^mice were subcutaneously inoculated with 1 x 10^5^ TC-1 cells and tumor growth was monitored until day 30 (D30) when at least 80% of the mice from each group were alive. Immune cell analyses of the tumor microenvironment were evaluated at day 21 after the tumor engraftment. **(B)** Frequency of CD45^+^ cells. **(C)** Frequency of CD11c^high^ MCH-II^high^ cells. **(D)** Frequency of CD8^+^ T cells. **(E)** Frequency of CD4^+^ T cells. **(F)** Frequency of FoxP3^+^ CD25^+^ T cells. Data from one experiment (n=9) were pooled and analyzed by unpaired t-test. **(G**, **H)** Effects of 1MT isoforms on the expression IL-6 by TC-1 cells. **(G)** Real-time PCR assay - Relative gene expression of IL-6 in TC-1 cells with or without 1mM D-1MT or DL-1MT treatment for 24h (n=3). **(H)** CBA assay - IL-6 release by TC-1 cells with or without exposure to 1mM D-1MT or DL-1MT for 24h (n=3). **I** Wild-type (WT) and IL-6^-/-^ mice were subcutaneously inoculated with 1 x 10^5^ TC-1 cells and vaccinated with two doses (D7 and D14) of gDE7 (30µg per animal). The experimental groups were followed for 60 days, but the “endpoint data” for each group was plotted up to the date when at least 80% of the mice were alive. **(J-L)** The antitumor effects of gDE7 in WT IL-6^-/-^mice were measured by **(J)** tumor volume (mm^3^), **(K)** percentage of mice survival and (**L**) percentage of tumor-free mice. **(M)** Frequency of circulating E7-specific CD8^+^ IFN-γ^+^/CD8^+^ T cells on day 21 (D21) after overnight *ex-vivo* stimulation of cells with the HPV-16 E7 Kb MHC class I-restricted immunodominant epitope peptide. **(B-F)** Data from one experiment (n=9) were pooled and analyzed by unpaired t-test. **(A**, **J-M)** data represent means ± SD from two independently performed experiments (n = 12) with comparable results and analyzed by ANOVA or by Kaplan-Meyer test (exclusively for survival assay). **(K)** One and four mice from WT + gDE7 and IL-6^-/-^+ gDE7 groups survived until day 60, respectively. (&) p <0.05, statistical significance of wild type (WT) group concerning all the others; (#) p <0.05, statistical significance of IL-6^-/-^+ gDE7 group concerning all the others; (*) p<0.05, (***) p<0.001, statistical significance of one experimental group concerning the other group. Regarding tumor volume graphs, the results were confirmed through multiple comparisons by Turkey’s test (three or four groups) or Sidak’s test (two groups), comparing each group mean with the other group mean at the same time point.

Next, we assessed how IL-6 affects tumor development (**Figure 3A**) and tumor-infiltrating immune cells (**Figures 3B-F**) (see **Figure 2E** for gate strategy) in IL-6^-/-^ mice engrafted with TC-1 cells. Tumor growth was significantly reduced in IL-6^-/-^ mice when compared to WT mice (**Figure 3A**). Furthermore, the frequency of immune cells (CD45^+^ cells) in the tumor microenvironment was increased in IL-6^-/-^ animals with a higher rate of intratumoral dendritic cells (DCs) compared to WT mice (**Figures 3B, C**), but no significant differences were observed in the frequencies of intratumoral CD8^+^ and CD4^+^ T lymphocytes, and Treg (**Figures 3D-F**). Notably, the transplanted TC-1 cells were capable of producing IL-6 (**Figures 3G, H**), underlining the importance of endogenous IL-6 on immune and stromal cells in the promotion of tumor growth. Following that, we investigated the influence of IL-6 on the antitumor effects of the gDE7 vaccination (**Figure 3I**). Immunotherapy reduces tumor development in IL-6^-/-^ mice (**Figure 3J**) with a significant improvement in survival but does not induce tumor remission (**Figures 3K, L**). Furthermore, no increase in the frequency of circulating E7-specific CD8^+^ IFN-^+^ T cells (**Figure 3M**) was seen at the time point analyzed (D21). These findings show that IL-6 promotes the growth of TC-1 cell proliferation and negatively impacts the efficacy of gDE7-based immunotherapy.

### IDO inhibition boosts the antitumor effects of gDE7 in IL-6^-/-^mice

**Figure 4:**
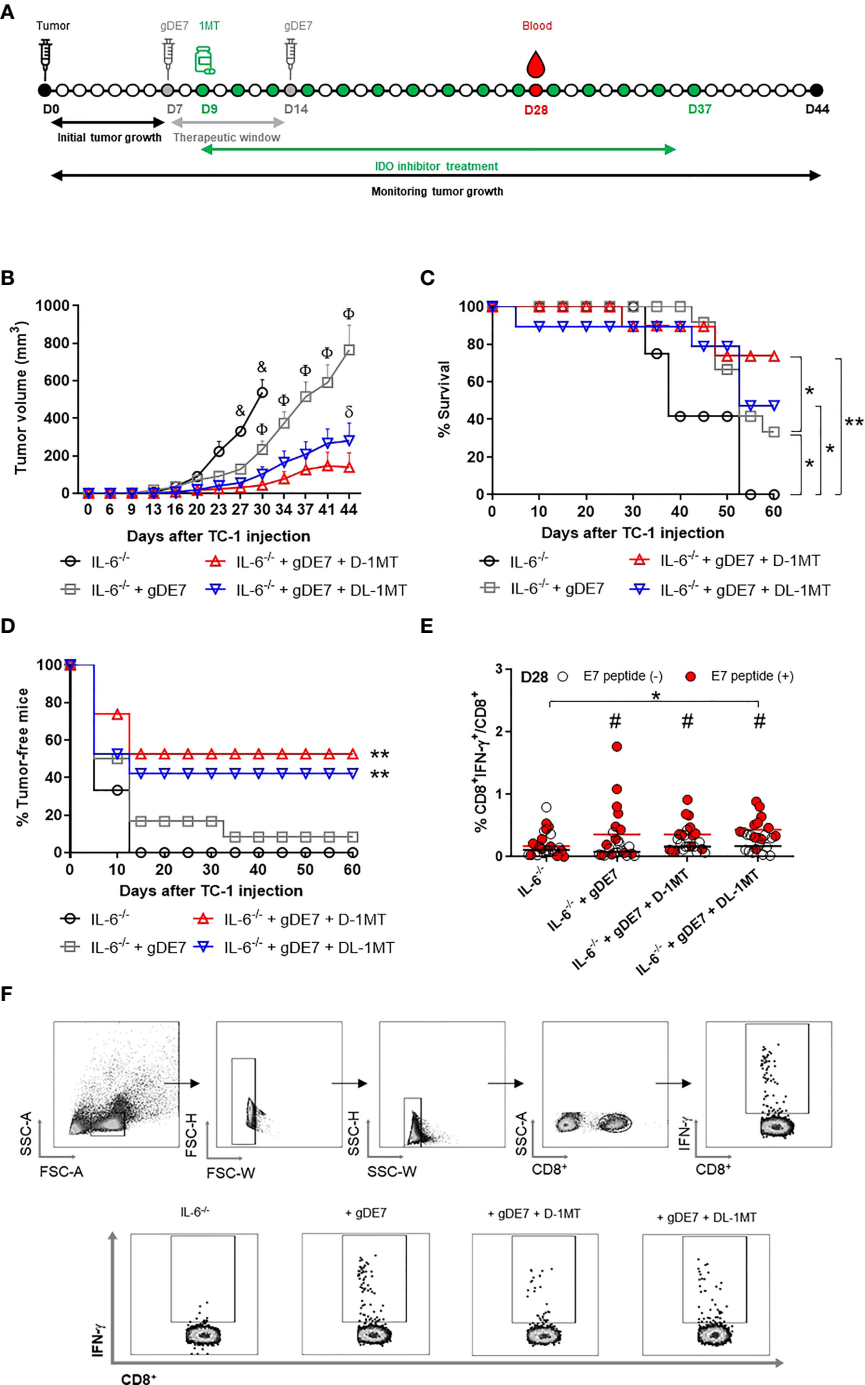
Lack of IL-6 combined with IDO inhibition augment immunotherapy control mediated by gDE7 on TC-1 cells engrafted in mice. **(A)** IL-6^-/-^mice were subcutaneously inoculated with 1 x 10^5^ TC-1 cells and vaccinated with two doses (D7 and D14) of gDE7 (30µg per animal). Two days after the first dose (D9), mice were treated with 1MT at a concentration of 10 mg/animal every other day for four weeks, until D37. The experimental groups were followed for 60 days, but the “endpoint data” for each group was plotted up to the date when at least 80% of the mice were alive. **(B-D)** Data represent means ± SD from two (groups IL-6^-/-^ and IL-6^-/-^ + gDE7) (n=6, total n=12) or three (groups IL-6^-/-^ + gDE7 + 1MT) (n=6 or 7, total n=19) independently performed experiments with comparable results and analyzed by ANOVA or by Kaplan-Meyer test (exclusively for survival assay). The antitumor effects of gDE7 combined with 1MT isoforms were followed by **(B)** tumor volume (mm^3^), **(C)** mice survival, and **(D)** presence of tumor-free mice. **(C)** Four, fourteen, and nine mice from IL-6^-/-^ + gDE7, IL-6^-/-^ + gDE7 + D-1MT, and IL-6^-/-^ + gDE7 + DL-1MT groups survived until day 60, respectively. **(E)** Frequency of circulating E7-specific CD8^+^ IFN-γ^+^/total CD8^+^ T cells on D28 after overnight *ex-vivo* stimulation of cells with the HPV-16 E7 Kb MHC class I-restricted immunodominant epitope peptide (n=12). **(F)** Gate strategy of circulating IFN-γ producing CD8^+^ T cells by flow cytometry. (&) p <0.05, statistical significance of IL-6^-/-^ control group concerning all the others; (Φ) p <0.05, statistical significance IL-6^-/-^+ gDE7 group concerning all the others; (δ) p <0.05, statistical significance of IL-6^-/-^+ gDE7 + DL-1MT group concerning all the others. (*) p<0.05, (**) p<0.01, statistical significance of one experimental group in comparison with the other groups. (#) p<0.05 represents the statistical significance of stimulated (red dots) versus non-stimulated (white dots) cells inside each experimental group. Regarding tumor volume graphs, the results were confirmed through multiple comparisons by Turkey’s test (three or four groups), comparing each group mean with the other group mean at the same time point.

To further understand the interplay of IL-6 and tryptophan metabolism in HPV-related tumors, we next evaluated the *in vitro* impact of IDO inhibitors on IL-6 expression in TC-1 cells. As indicated in **Figure 3**, culturing TC-1 cells in the presence of D-1MT or DL-1MT significantly reduced IL-6 gene expression and cytokine release (**Figures 3G, H**). IL-6^-/-^ tumor-bearing mice were immunized with gDE7 and treated with IDO inhibitors following the “every other day” regimen (**Figure 4A**). In comparison to the gDE7-treated group, IL-6^-/-^ mice vaccinated with gDE7 and treated with D-1MT or DL-1MT showed significantly decreased tumor mass (**Figures 4B-D**). IL-6^-/-^ mice treated with gDE7 and D-1MT had a 73% survival rate and 52% remained tumor-free till the end of the observation period (D60), whereas animals treated with gDE7 and DL-1MT had a 52% survival rate and 47% were tumor-free. In contrast, IL6^-/-^ mice treated only with gDE7 showed a 33% survival rate and 10% remained tumor-free at D60. Importantly, comparing WT mice with IL-6 KO mice, both treated with the combination of gDE7 + IDO inhibitors, we observed a significantly decreased in tumor growth in IL-6 defective mice, especially when treated with D-1MT (**Supplementary Figure S1**). Furthermore, at D28, higher numbers of circulatory E7-specific CD8^+^ IFN-γ^+^ T cells were observed in mice that received the combined therapy (**Figures 4E, F**). Importantly, we opted to assess CD8^+^-specific T cells on D28 because when we previously evaluated this cell population in the mice blood on D21, we found no changes between the control and the gDE7-vaccinated groups (**Figure 3M**). Taken together, the current findings show that, in IL-6^-/-^mice, the combination of gDE7 with 1MT isoforms boosts the antitumor immunity and highlights the role of IDO and IL-6 on the growth of TC-1 cells.

### Lack of IL-6 expression and IDO inhibition enhances activation of intratumoral effector immune cells and reduces immune suppressive cells in gDE7 vaccinated mice

**Figure 5:**
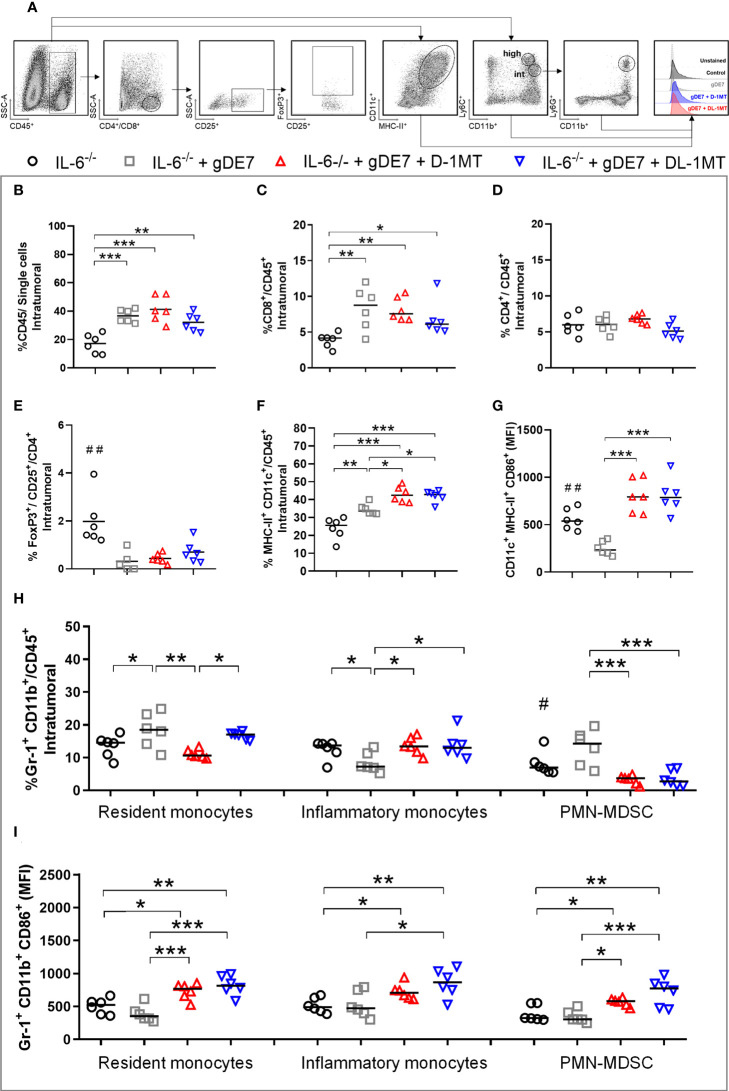
IDO inhibition increases activation of intratumoral DCs and decreases immune suppressive PMN-MDSC in tumor microenvironment of IL-6-^/^- mice immunized with gDE7. **(A)** Gating strategy for immune cell analyses of the tumor microenvironment, evaluated at day 21 after the tumor engraftment. Cells were gated by the expression of CD45^+^ and successively analyzed according to the expression of CD8^+^ (CD8^+^ T lymphocytes), CD4^+^ (CD4^+^ T lymphocytes) followed by CD25^+^ and FoxP3^+^ CD25^+^ (T regulatory cells); CD11c^high^ MCH-II^high^ (dendritic cells), CD11bint Ly6Cint Gr1^-^ (resident monocytes), CD11b^int^ Ly6C^high^ Ly6G^-^ (inflammatory monocytes) or CD11b^high^ Ly6C^int^ Ly6G^+^ (PMN-MDSC). Antigen-presenting cells were considered activated by the expression of the co-stimulatory molecule CD86, analyzed by the median of fluorescence intensity (MFI) in the gated subsets cells. **(B)** Frequencies of CD45^+^ cells. **(C)** Frequencies of CD8^+^ cells. **(D)** Frequencies of CD4^+^ cells. **(E)** Frequency of FoxP3^+^ CD25^+^ CD4^+^ cells. **(F)** Frequencies of CD11c^high^ MCH-II^high^ cells. **(G)** CD86 MFI in CD11c^high^ MCH-II^high^ cells. **(H)** Frequencies of Gr1^+^ (Ly6C^+^/Ly6G^-^ or Ly6C^-^/Ly6G^+^) CD11b^+^ cells. **(I)** CD86 MFI in Gr1^+^ CD11b^+^ subsets cells. Data representative of two independently performed experiments (n=6). Statistical significance: (*) p<0.05, (**) p<0.01, (***) p<0.001 by ANOVA. (#) p<0.05 and (##) p<0.01 represent the statistical significance of control group versus other groups.

We next investigate the tumor-infiltrating immune cells population (time point analyzed - D21) to determine the immunological mechanism behind the tumor rejection outcome obtained in IL-6 deficient mice by our therapeutic approach (**Figure 5A**). Mice immunized with gDE7 and those also treated with IDO inhibitors (D-1MT or DL-1MT) had higher rates of intratumoral CD45^+^ (**Figure 5B**) and CD8^+^ T cells (**Figure 5C**) than non-immunized mice. Interestingly, despite CD4^+^ T-cell population was similar in all experimental groups (**Figure 5D**), immunization with gDE7 with or without IDO inhibitors decreased the frequency of intratumoral Treg cells (**Figure 5E**). Regarding myeloid cells, immunization of IL-6^-/-^ mice with gDE7 enhanced DC migration into the tumor microenvironment (**Figure 5F**). In addition, the adjuvant treatments with D-1MT or DL-1MT increased the frequencies of activated DCs in the tumor microenvironment (**Figures 5F, G**). Moreover, mice treated with gDE7 and 1MT isoforms showed increased activation of resident and inflammatory monocytes (**Figures 5H, I**). Notably, the combined treatment of gDE7 and D-1MT or DL-1MT substantially reduced the frequency of intratumoral PMN-MDSC when compared to mice treated only with gDE7 (**Figure 5H**). Furthermore, the combination treatment with D-1MT or DL-1MT promoted upregulating of CD86 of PMN-MDSC (**Figure 5I**). Unfortunately, due to the small tumor volume, it was not possible to assess the E7-specific CD8^+^ IFN-γ^+^ T cells population in the tumor microenvironment. These findings further support the role of IL-6 and IDO in the immunomodulation promoted by gDE7 and underline the relevance of multi-target therapeutic strategies for successful antitumor immunotherapy.

## Discussion

Our research explored the possible association between IL-6 and IDO1 in the progress of HPV-related tumors, as well as their influence on a specific cancer immunotherapy strategy. The experimental approach aimed to circumvent three major concerns in HPV-related tumors: systemic and local high expression of IL-6 and IDO as well as activation of effector immune cells. To address this goal, we employed the well-known HPV-16 TC-1 tumor mouse model, which expresses IL-6 (30) and IDO (27), to understand the impact of these factors during gDE7-based immunotherapy. The main findings of the study were: 1) IL-6 impacts the *in vivo* TC-1 cell tumor development and this feature depends on IL-6 expression by leukocytes and stromal cells, not by tumor cells; 2) in IL-6^-/-^ mice, gDE7 treatment enables partial tumor mass control, nonetheless, only with IDO inhibition gDE7-immunized mice could boost immune responses and more efficiently eradicate tumor cells; 3) in the absence of IL-6, the adjuvanticity of 1MT isoforms were essential to increase the efficacy of the gDE7 vaccine leading to increased frequencies and activation of antigen-presenting cells in the tumor microenvironment and control of intratumoral PMN-MDSC and Treg expansion.

The vaccines based on the fusion of HPV-16 E7 oncoprotein and the HSV-1 glycoprotein D, either protein- or DNA-based, have been shown to potentiate immune responses capable of blocking inhibitory signals mediated by the B and T lymphocyte attenuator (BTLA) co-signaling protein by competitive binding inhibition with herpesvirus entry mediator (HVEM) receptor (31, 32). In addition, HSV-1 gD protein delivers target antigen and promotes direct activation of a specific DCs subset specialized in cross-presentation leading to efficient activation of CD8^+^ T cell-dependent antitumor responses (25). The therapeutic antitumor efficacy of gDE7-based vaccines could be enhanced by combination with different adjuvant procedures, including administration by electroporation (33), combined treatments with gemcitabine (34) and cisplatin (26), the addition of poly(I:C) (25, 35), co-expression of IL-2 (36) or IL-10 receptor (37), adsorption to *Bacillus subtilis* spores (38), and combination with metabolic adjuvants such as IDO inhibitors and melatonin (27). Recently, a novel antibody-based vaccine platform was designed to deliver E7 oncoprotein to DEC205^+^ dendritic cells (αDEC205-E7 mAb) (39). Although gDE7-based vaccines showed outstanding anticancer effects, the effectiveness decreased when tumors achieved an advanced growth stage due to immunosuppressive mechanisms elicited by tumor cells (27, 34, 37). Indeed coadministration of DNA vaccines encoding gDE7 and IL-10 receptors has been shown to halt tumor-induced immune suppressive cells (MDSC) and enhance strong tumor-specific CD8^+^ T-cell response leading to better control of tumors at advanced growth stages (37). Notably, IL-10^-/-^ mice develop TC-1 cell-derived tumors at faster rates with a significant enhancement of IL-6 and numbers of intratumoral MDSC concerning WT mice. Indeed, previous experimental evidence demonstrated that the use of an anti-IL-6 receptor monoclonal antibody controlled tumor growth and expansion of intratumoral MDSC (40).

Among the oncology biomarkers explored in HPV-related cancers, IL-6 stands out as a predictor of tumor development and immunosuppression. (9, 41–43). High expression of IL-6 in both tumor cells and surrounding tissues has been found in patients with HPV-16 and 18 infections (8, 42, 44). It is widely assumed that the positive regulation of IL-6 in HPV-related pathologies relies on STAT3 signaling (15, 43). Interestingly, the STAT3/IL-6 axis is assumed to regulate the constitutive expression of IDO1 in tumor cells (16), leading to the hypothesis that this axis could regulate the high-level expression of IDO1 in the tumor microenvironment and adjacent tissues of HPV-related tumors (27, 45–48), since both molecules are co-expressed in TC-1 cell mouse model or cervical cancer patients. Indeed, IL-6 and IDO have been linked to poor treatment outcomes, tumor recurrence, and aggressive tumor progression in breast cancer (49), nasopharyngeal carcinoma (50), and prostate cancer (51) patients. Therefore, the study of these two immunosuppressive molecules is important not only for HPV-related tumor, but also for other tumor types. Considering that immunometabolism has emerged as a central element in cancer therapy (52, 53), we previously explored the combination of gDE7 with IDO inhibitors and melatonin, which promoted synergic antitumor effects drawing attention to the relevance of multi-target therapeutic approaches (27). Focusing on continuing this study and further understanding the IDO/IL6 axis in HPV-related tumors, in the present study we investigated the outcomes of gDE7 immunization in IL-6^-/-^ mice, treated or not with 1MT isoforms, based on the hypothesis that targeting IDO and IL-6 could augment the immunotherapeutic effects. Aligning with our findings, the impact of IL-6 in HPV-related tumors has been previously demonstrated with IL-6^-/-^ mice and by blocking of IL-6 with specific inhibitors (43). Here we saw a considerable reduction in tumor development when the IL-6^-/-^ mice were immunized with gDE7. This phenomenon could be related to the specific tumor signature since IL-6 deficiency did not affect esophageal tumorigenesis (54).

IL-6 signaling can be targeted in a variety of ways, including the use of anti-IL-6 (siltuximab) or IL-6R (tocilizumab) monoclonal antibodies, which have both been extensively studied in different experimental tumor models as well as in clinical trials (55). IL-6 inhibition combined with other chemotherapeutic drugs, radiation, and targeted therapies significantly increased the clinical therapeutic gain in various cancer types (56, 57). In this concern, the inhibition of IDO1 can trigger an IL-6-dependent toxic inflammation in mice, which can be reduced by anti-IL-6 antibodies (58). Indeed, the combination of different treatments with multifactorial target mechanisms may pave the way for the generation of new and more effective cancer therapies. Focusing on cancer metabolism, we previously observed the impact of IDO1 on the efficacy of gDE7 immunization, with the complete rejection of TC-1 tumors in IDO1-deficient mice (27). Taking this finding into account, and knowing that giving oral IDO inhibitors every other day partially protects WT mice immunized with gDE7 (27), we sought to find out if oral administration of 1MT isoforms to WT mice every day would enable tumor clearance in response to gDE7 treatment. This approach leads to toxic side effects that impaired the antitumor responses conferred by gDE7. Similar findings were obtained in an experimental HPV-related head and neck tumor model using tumor cells derived from murine oropharyngeal epithelial cells expressing HPV16 E6/E7 (59). However, in a glioblastoma mouse model (60) and a lung mouse model (61), daily oral administration of 1MT isoforms improved therapeutic outcomes.

Targeting IDO1-induced immunosuppressive mechanisms could represent a double-edged sword, since inhibiting IDO1 as a monotherapy could also lead to increased tumor development (27, 62). Clinical development efforts now encompass the combination of IDO inhibitors with immunotherapies. Positive clinical outcomes were achieved when IDO inhibitors were used in combination with sipuleucel-T (NCT01560923), DC-based vaccine (NCT01042535), and pembrolizumab (63), implying that IDO inhibition has a significant therapeutic value when combined with other therapeutic procedures. Regarding gDE7 immunotherapy, we observed a similar adjuvanticity performance of DL-1MT and D-1MT, worth mentioning that D-1MT is presently undergoing 17 clinical trials. Both 1MT isomers lead to increased gDE7-mediated antitumor protection in WT mice, but only the combination of gDE7 with IDO inhibitors in the absence of IL-6 afforded more efficient tumor cell eradication. Importantly, TC-1 cells were the exclusive IL-6 source in the model suggesting that the therapeutic efficacy is selective when targeting IL-6 on immune and stromal cells rather than on the tumor cells. The immunotherapeutic efficacy of the proposed vaccine approach (gDE7 + IL6^-/-^ + 1MT) relied on the immune-cellular profile of the tumor microenvironment, including activation of myeloid cells and reduction of PMN-MDSC and Treg. Supporting our data, IL-6 is involved in the differentiation and expansion of MDSCs, which can inhibit T-cell *via* multiple molecular mechanisms (64), and 1MT effectively reverses the recruitment of tumor-infiltrating MDSCs induced by IDO1 (65). Similarly, as previously noted, CD11b^+^Ly6G^+^ myeloid cells represent a major source of IDO in the tumor microenvironment (58). Treg cells are also involved in the role of IDO1-induced immunosuppressive mechanisms that promotes cancer cell survival (20). Indeed, higher frequency of CD4^+^CD25^+^FoxP3^+^ T cells are associated with IDO expression in immunological and stromal cells (66, 67). In this concern and corrobotarting or data, the inhibition of IDO by 1MT attenuates Treg cells differentiation and expansion (67, 68).

Concerning the therapeutic effectiveness, DCs are required for immunotherapy-driven tumor relapse control (25, 26, 38). Our current data with the IL6^-/-^ mouse model demonstrated the relevance of 1MT adjuvanticity in boosting DCs in the tumor microenvironment. Indeed, cooperativity between orally-delivered 1MT and subcutaneous administration of gDE7 in IL-6^-/-^ mice induced tumor rejection and DC activation. Interestingly, we observed an increased frequency of DCs in the tumor microenvironment of IL-6^-/-^ mice, but not CD4^+^ T cells or CD8^+^ T cells at the time point assessed. Corroborating our data, an increased percentage of DCs was observed in IL-6^-/-^ mice implies that IL-6 hinders DC maturation *in vivo* with negative outcomes for DC-mediated T cell activation (14). Notably, IL-6^-/-^ DCs retained the ability to generate functional CD8^+^ T effectors and memory cells (69). In this concern, the IL-6 signaling cascade was shown to inhibit the expression of major MHC-II and CD86 molecules on the surfaces of DCs *in vivo*, resulting in the delay of cancer-related antigen presentation (70, 71). Moreover, the dysfunction of DC also attenuates CD4^+^ T-cell-mediated antitumor immunity responses, and inhibition of IL-6 reduces tumor growth by restoring T-cell activity in tumor-bearing mice (72, 73). The tumor-driven immunosuppression of DCs could also rely on IDO expression (74). Remarkably, in the TC-1 tumor mouse model, there is a substantial increase in tumor-infiltration of IDO-expressing DCs, macrophages, and monocytes during tumor development, which contributes to the immunosuppressive cellular microenvironment (27). Although IDO inhibitors did not increase intratumoral CD8^+^ T cells in vaccinated mice, concomitant targeting of IL-6 and IDO promoted efficient induction of tumor-infiltrating DCs. Notably, cellular analyses indicated that only gDE7 combined with 1MT increased tumor infiltration of monocytic and myeloid antigen-presenting cells expressing higher levels of CD86, an important T-cell costimulatory molecule. Therefore, one possible hypothesis of the observed antitumor effects may rely on the fact that 1MT can reverse the T cell suppressive phenotype induced by IDO-expressing murine DCs promoting efficient antigen presentation and T cell proliferation (62).

In the era of immuno-oncology, the search for prognostic markers to expand the use of the immunotherapeutic approach may be a key step to improving the outcome of presently available cancer treatments. The contextual study presented here has allowed us to demonstrate how anti-IDO/IL-6 therapies may contribute to future successful treatments and open perspectives for the development of alternative options for the treatment of HPV-related tumors.

## Data availability statement

The original contributions presented in the study are included in the article/**Supplementary Material**. Further inquiries can be directed to the corresponding author.

## Ethics statement

Mice experiments were performed under approved protocols by the ethics committee for animal experimentation from Instituto de Ciências Biomédicas da USP, protocol number CEUA 8572030918.

## Author contributions

RLP, AM and LF conceptualized the study. BP and LA designed the vaccine platform. RLP, P.C.S., and RP carried out tumor cell engraftments and performed immunizations. RLP, PS, RP, BP, JS, LA, MS, KR, NS and AM performed acquisition, analysis, and interpretation of data. All authors participated in the interpretation of the results. RLP, AM and LF wrote the paper. AM and LF were responsible for raising funding for the study. AM supervised the study. All authors contributed to the article and approved the submitted version.

## Funding

This work was supported by Fundação de Amparo à Pesquisa do Estado de São Paulo (FAPESP), Coordenação de Aperfeiçoamento de Pessoal de Nível Superior (CAPES), and Conselho Nacional de Desenvolvimento Científico e Tecnológico (CNPq). RLP was fellow supported by FAPESP 2017/25544-4; AM was supported by FAPESP, grant 2015/16505-0 and fellow 2016/00708-1; L.C.S.F. was supported by the CNPq, grant 520931/1996-3 and by FAPESP grant number 2016/20045-7; PS was fellow supported by CNPq 148913/2016-4; RP was fellow supported by Programa Institucional de Bolsas de Iniciação Científica (PIBIC)/CNPq; BP was fellow supported by FAPESP 2017/21358-1; JS was fellow supported by FAPESP 2016/11594-7; LA was fellow supported by FAPESP 2013/15360-2 and 2018/08502-9; MS was fellow supported by FAPESP 2016/11397-7; KR was fellow supported by CAPES 146598/2016-4 and by FAPESP 2021/03326-0; NS was fellow supported by FAPESP 2016/14344-1.

## Acknowledgments

The authors would like to thank Dr. T. C. Wu from John Hopkins University, Baltimore, MD, USA for kindly providing the TC-1 cell line in 2002, and Dr. Ana Paula Lepique from the Department of Immunology, Instituto de Ciências Biomédicas, Universidade de São Paulo, for kindly providing the IL-6 knockout (IL-6^-/-^) mice. The authors are especially grateful to Mr. Eduardo Gimenes Martins, from the Department of Microbiology, Instituto de Ciências Biomédicas, Universidade de São Paulo, for the technical and administrative support related to the execution of this project.

## Conflict of interest

BP and LA hold ownership interest, including patents, in ImunoTera Soluções Terapêuticas Ltda.

The remaining authors declare that the research was conducted in the absence of any commercial or financial relationships that could be construed as a potential conflict of interest.

## Publisher’s note

All claims expressed in this article are solely those of the authors and do not necessarily represent those of their affiliated organizations, or those of the publisher, the editors and the reviewers. Any product that may be evaluated in this article, or claim that may be made by its manufacturer, is not guaranteed or endorsed by the publisher.
