# Supplementary material for: Interleukin-6 and indoleamine-2,3-dioxygenase as potential adjuvant targets for Papillomavirus-related tumors immunotherapy
Source: Front Immunol. 2022 Nov 3;13:1005937. doi: 10.3389/fimmu.2022.1005937 (PMC9668887; doi:10.3389/fimmu.2022.1005937)
Supplement: Supplementary file 1 [file DataSheet_1.pdf]

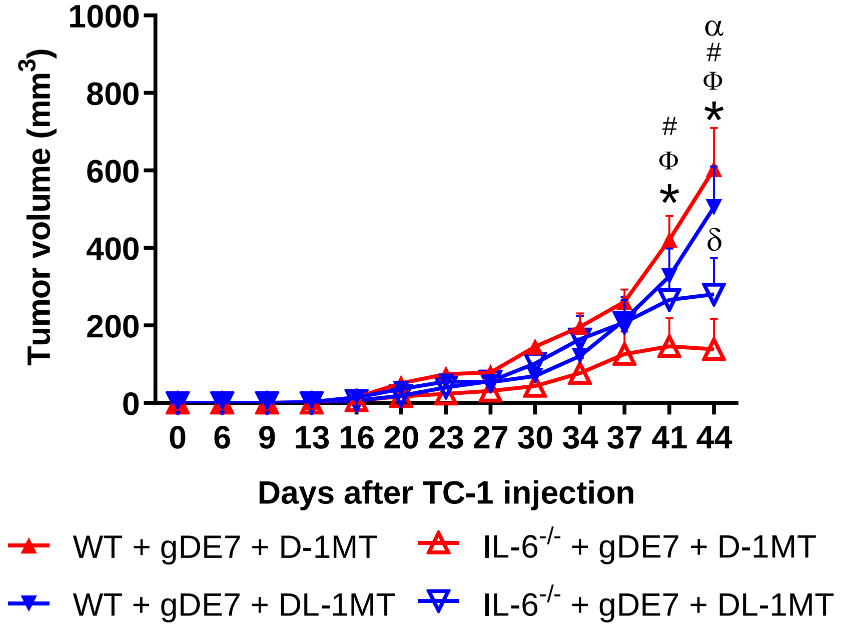

Figure S1. Lack of IL-6 combined with IDO inhibition augment immunotherapy control mediated by gDE7 on TC-1 cells engrafted in mice. WT and IL-6$^{-/-}$ mice were subcutaneously inoculated with 1 x 10^{5} TC-1 cells and vaccinated with two doses (D7 and D14) of gDE7 (30µg per animal). Two days after the first dose (D9), mice were treated with 1MT at a concentration of 10 mg/animal every other day for four weeks, until D37. The experimental groups were followed for 60 days, but the "endpoint data" for each group was plotted up to the date when at least 80% of the mice were alive. Data represent means ± SD from two (groups WT + gDE7 + 1MT) (n=5, total n=10) or three (groups IL-6$^{-/-}$ + gDE7 + 1MT) (n=6 or 7, total n=19) independently performed experiments with comparable results and analyzed by Two-Way ANOVA and the results were confirmed through multiple comparisons by Turkey's test. (*) $p < 0.05$, statistical significance of WT + gDE7 + D-1MT concerning IL-6$^{-/-}$ + gDE7 + D-1MT; (Φ) $p < 0.05$, statistical significance of WT + gDE7 + D-1MT concerning IL-6$^{-/-}$ + gDE7 + DL-1MT; (#) $p < 0.05$, statistical significance of WT + gDE7 + DL-1MT concerning IL-6$^{-/-}$ + gDE7 + D-1MT; (α) $p < 0.05$, statistical significance of WT + gDE7 + DL-1MT concerning IL-6$^{-/-}$ + gDE7 + DL-1MT; (δ) $p < 0.05$, statistical significance of IL-6$^{-/-}$+ gDE7 + D-1MT concerning IL-6$^{-/-}$ + gDE7 + DL-1MT.
